# Short-time speaker verification with different speaking style utterances

**DOI:** 10.1371/journal.pone.0241809

**Published:** 2020-11-11

**Authors:** Hongwei Mao, Yan Shi, Yue Liu, Linqiang Wei, Yijie Li, Yanhua Long

**Affiliations:** 1 SHNU-Unisound Joint Laboratory of Natural Human-Computer Interaction, Shanghai Normal University, Shanghai, China; 2 Unisound AI Technology Co., Ltd., Beijing, China; University of Kent, UNITED KINGDOM

## Abstract

In recent years, great progress has been made in the technical aspects of automatic speaker verification (ASV). However, the promotion of ASV technology is still a very challenging issue, because most technologies are still very sensitive to new, unknown and spoofing conditions. Most previous studies focused on extracting target speaker information from natural speech. This paper aims to design a new ASV corpus with multi-speaking styles and investigate the ASV robustness to these different speaking styles. We first release this corpus in the Zenodo website for public research, in which each speaker has several text-dependent and text-independent singing, humming and normal reading speech utterances. Then, we investigate the speaker discrimination of each speaking style in the feature space. Furthermore, the intra and inter-speaker variabilities in each different speaking style and cross-speaking styles are investigated in both text-dependent and text-independent ASV tasks. Conventional Gaussian Mixture Model (GMM), and the state-of-the-art x-vector are used to build ASV systems. Experimental results show that the voiceprint information in humming and singing speech are more distinguishable than that in normal reading speech for conventional ASV systems. Furthermore, we find that combing the three speaking styles can significantly improve the x-vector based ASV system, even when only limited gains are obtained by conventional GMM-based systems.

## Introduction

Automatic speaker verification (ASV) is using the speaker’s speech signal to extract the identity of the speaker [1, 2]. It is an important biometric recognition technology, which is widely used in access control security authentication, personalized services, etc. In recent years, the ASV technology has achieved great success. Many session, channel compensation methods [3–5], new front-end [6, 7], framework and speaker modeling algorithms [8–12] have been proposed. However, in real-world ASV applications, most technologies are still vulnerable to new domain, intra and inter-speaker variability, spoofing attacks, etc. The performances of ASV systems will be significantly degraded when there is mismatch between registration and test speech utterances.

For improving the ASV robustness in real applications, most previous works focused on extracting the speaker characteristics from normal natural speech. Such as the read speech, spontaneous or contextual speech [1]. However, with the rapid development of intelligence speech technologies, more and more ASV-related applications are coming into most people’s life, such as personalized accessing of WeChat, QQ accounts, etc. Users start to worry about the security of ASV systems, they worry about their voiceprint characteristics are not unique enough, because they know that using nowadays’ speech technologies, their normal speech is easy to be imitated, replayed or synthesized [13], etc. Therefore, some users may choose to use their special speaking style during the ASV target speaker enrollment stage, such as humming, singing, speaking with emotion, or whispering, etc.

Actually, it is challenging to develop a general robust ASV system to handle flexible speaking style in real applications. For the speaker verification using speech with special speaking styles, very limited previous works can be found in the literature. In [14], the authors analyzed the ASV system behaviors using speech with low, normal and high vocal efforts. They found that vocal effort variation can significantly degrade system performance. And in [15], the authors found that in contrast to high-effort read speech, high-effort oration shares characteristics with conversational and interview styles. In [16, 17], the authors examined the human and machine speaker discrimination ability between read sentences and speech directed towards pets. They also found that the speaking style-mismatch can result in significant ASV performance reduction. Work in [18, 19] investigated building speaker verification systems based on whispered and normal speech. They focused on extracting new features to improve the system performances, bottleneck, spectral and modulation spectral features were proposed in [18], while [19] proposed using auditory inspired amplitude modulation spectrum and cepstral features. For the ASV with emotional speech, work [20] found that speech with various emotions aggravates the verification performance because the articulating styles of certain emotions can create intense intra-speaker vocal variability. Other related works were using the humming and singing speech to extract target speaker identity information. Such as, [21, 22] evaluated the effects of convention acoustical features (MFCC, PLP and LPCC) on humming ASV speech; [23] proposed an variable length teager energy based MFCC to identify speakers from their hum; [24] built ASV systems on the humming, singing and normal reading speech separately, they found that the humming sounds are better than speech and singing for capturing speaker-specific characteristics. All of these previous works provide a good reference for studying speaker recognition with different speaking styles. However, most of these works were based on the conventional Gaussian Mixture Model (GMM) speaker verification framework, comparable results by using the state-of-the-art deep neural network were extremely limited. Moreover, previous experiments focused more on the single-speaking style speech [18, 20, 21, 25], but in real ASV applications, the ASV testing with cross-speaking style is also very common. In addition, except for our previous work in [26], we have not found any other works addressed the ASV using multi-speaking style Mandarin speech.

In our previous work [26], we released a Mandarin speech ASV corpus with both the reading and singing style speech(RSS). Based on this corpus, we first compared the differences between reading and singing speech in two acoustic feature spaces, then, the GMM, Dynamic Time Warping, and the state-of-the-art x-vector ASV systems were built to compare the effectiveness of each speaking style speech. However, from the preliminary experimental results of short-time text-dependent ASV tasks, we found that the ASV performances from singing speech were only slightly better than the ones obtained from reading speech. From the detail analysis in [26], we knew that this was because all of the speakers are very familiar to the singing songs in RSS corpus, the melody of these original songs guide the speaker to sing with a similar singing style. Therefore, the discriminative information between different speakers in RSS is significantly reduced.

In order to remedy for the deficiency of our previous work on RSS, in this study, we first design and release a new Mandarin multi-speaking style ASV corpus-“RSH”. Compared with RSS, this corpus focuses on recording a larger corpus between reading, singing and humming speech using each speaker’s personalized password text. During recording, the speakers can read, sing and hum using their own styles. Then, based on the RSH corpus, we first investigate the speaker discriminations in the feature space using t-SNE [27], then we not only examine the target speaker’s discrimination between each single-speaking style, but also investigate the behavior of ASV systems with cross-speaking style testing. Both the text-independent (TI) and text-dependent(TD) short-time ASV tasks are constructed in this study. The conventional GMM and state-of-the-art x-vector [9] are used for our speaker modeling. Experimental results show that the voiceprint information in the singing speech and humming are more distinguishable than the one in the natural reading speech both for the TI and TD short-time ASV tasks. Furthermore, we have released the RSH corpus on the Zenodo website https://zenodo.org/record/3816618 and put our system implementation code in the github repository https://github.com/Moonmore/Speaker-recognition-based-on-RSH for public research. And from these two websites, we find that our previous RSS corpus has been attracted many researcher’s attention, it has been downloaded more than 150 times until now. We hope this new release can attract more research attention on the multi-speaking style ASV task.

## RSH corpus

### Basic information

To remedy the deficiency of our previous RSS corpus [26], a larger new corpus is designed for this study. Unlike the RSS with only singing and reading speech, this corpus includes three speaking-style utterances, the normal reading, singing and the humming. For simplicity, we name this new corpus as “RSH”. A detail description of RSH is shown in Table 1. It consists of 46 speakers, including 20 male and 26 female undergraduate students. As our motivation is to investigate the effectiveness of multi-speaking style speech for speaker verification, only 19 to 24 year old students are selected as our target speakers during the corpus recording. All these students participated in the corpus recording task through the open recruitment process. All participants were informed and agreed to the recording task and signed a written informed consent form.

**Table 1:** RSH corpus description.

Item	RSH Details
Recording software	Audition
Language	Mandarin
Text	10 daily used phrase or sentence for all speakers, 1 personalized unique text for each speaker
Environment	quiet lab environment
Format	16,000 Hz, 16 bit, 1 channel
Speaker	46 undergraduate students(20 male,26 female)
Microphone	common laptop built-in microphone
Format	16,000 Hz, 16 bit, 1 channel
Biometric signal	singing, humming and reading speech

To eliminate the melody guidance effect of the original songs in the previous RSS corpus, in this new corpus, the phrases or short sentences that are frequently used in students’ daily life are selected as the recording text. Each text contains 4-9 words. During the recording, the speakers are required to sings, hums and reads the given text in their personal special styles. All of the recordings are collected in a quiet laboratory environment using a normal notebook built-in microphone. We use “audition” as our recording and editing software. To create reasonable comparative experiments with three styles speech recordings, for each speaker and each text, we record it three times per speaking style in two days at different time. Each recording is about 1 to 2 seconds and formatted as 16kHz, 16bit WAV file. We choose this specific format because it is more generally used in most human-machine interaction applications. Moreover, the 16kHz WAV format is also normally used in automatic speech recognition applications, it will be better to keep the same speech input setup to make things compatible in real-world applications. All of the recordings are in Mandarin. (Our speech data collection was reviewed and approved by the ethics committee of the College of Information, Mechanical and Electrical Engineering, Shanghai Normal University before this study began.)

### ASV task construction

In this study, we focus on exploring how the multi-speaking style speech affects both the text-dependent and text-independent ASV tasks. Table 2 shows the details of ASV tasks we designed based on the “RSH” corpus. Since for each speaking style, each speaker has three short recordings with the same text, we randomly select one of them for the target speaker registration, and one of the other two recordings is taken as the test data. There is no overlap between the registration and test speech recordings. In the end, for the TD-based ASV task, we select a total of 460 (46 × 10) speaker enrollment data recordings, including 200 for males and 260 for females. Each recording is taken to train one target speaker model.

**Table 2:** Details of text-dependent and text-independent short-time ASV tasks.

Task	Target Speakers	Test Segments	Target Trials	Nontarget Trials
TD-GD	460	460	200 male,260 female	3800 male,6500 female
TD-GI	460	460	460	20700
TI-GD	460	460	2000 male,2600 female	38000 male,65000 female
TI-GI	460	460	4600	207000

Besides investigating the ASV effectiveness of multi-speaking style speech with text variation, we also expect to explore the inter and intra-speaker variabilities of multi-style speech in different gender, because observing the performance differences between gender can help us to better understand the speech production differences between two genders. Therefore, as shown in Table 2, we create two TD and TI ASV tasks separately. “GD” and “GI” represent the gender-dependent and gender-independent respectively. Considering the 10 shared texts mentioned in Table 1, so in the TD-GD task, we generate 20 × 10 male, 26 × 10 female target test trials, and 20 × 19 × 10 = 3800 non-target test trials. In the TD-GI task, we have 46 × 10 = 460 target test trials, and 46 × 45 × 10 = 20700 non-target test trials. However, in the TI-based tasks, we have the 460 × 10 = 4600 target test trials, and 460 × 45 × 10 = 207000 non-target test trials.

In the experiments, all of the ASV tasks are created in the same way as the TD and TI based tasks in Table 2, including the tasks for each single-speaking style, or the cross-testing tasks with mismatched enrollment and test speaking styles. As shown in Table 2, the target speaker models are the same for both the TD and TI tasks, the difference between TD and TI tasks are their evaluation (test) trials, these test trials are designed as “text-dependent” and “text-independent” respectively. All experiments are performed on the above corpus and experimental configuration. More details about RSH data and experimental configuration files, please refer to the Zenodo website and github repository mentioned in the introduction.

## Speaker discrimination in feature space

Before building ASV real systems, we first investigate the speaker discrimination ability of different speaking-style recordings in the feature space. We applied the t-SNE [27] to project the 60-dimensional MFCC features (please refer to section for details) to a 2-dimensional space for visualization.

**Fig 1:**
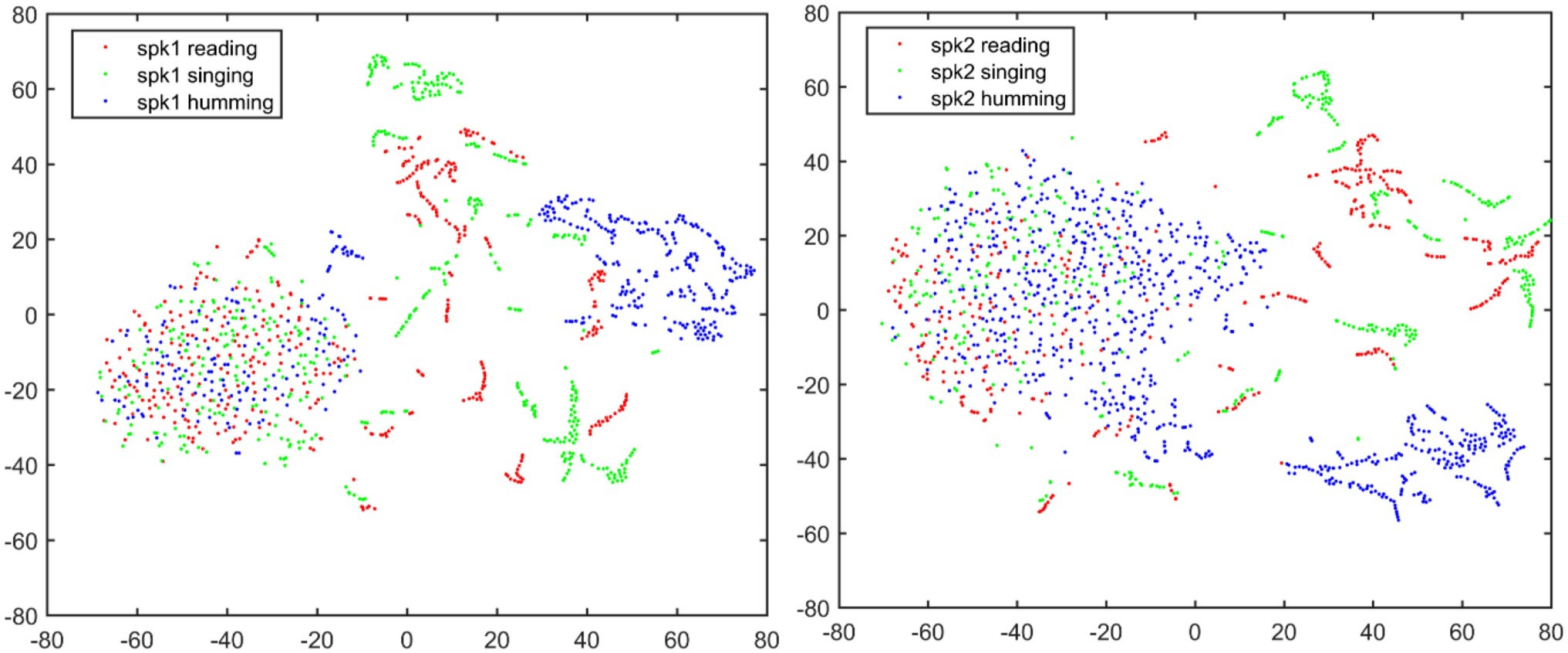
t-SNE visualization of two speakers’ features under text-dependent condition. All the texts are the same for two speakers.

**Fig 2:**
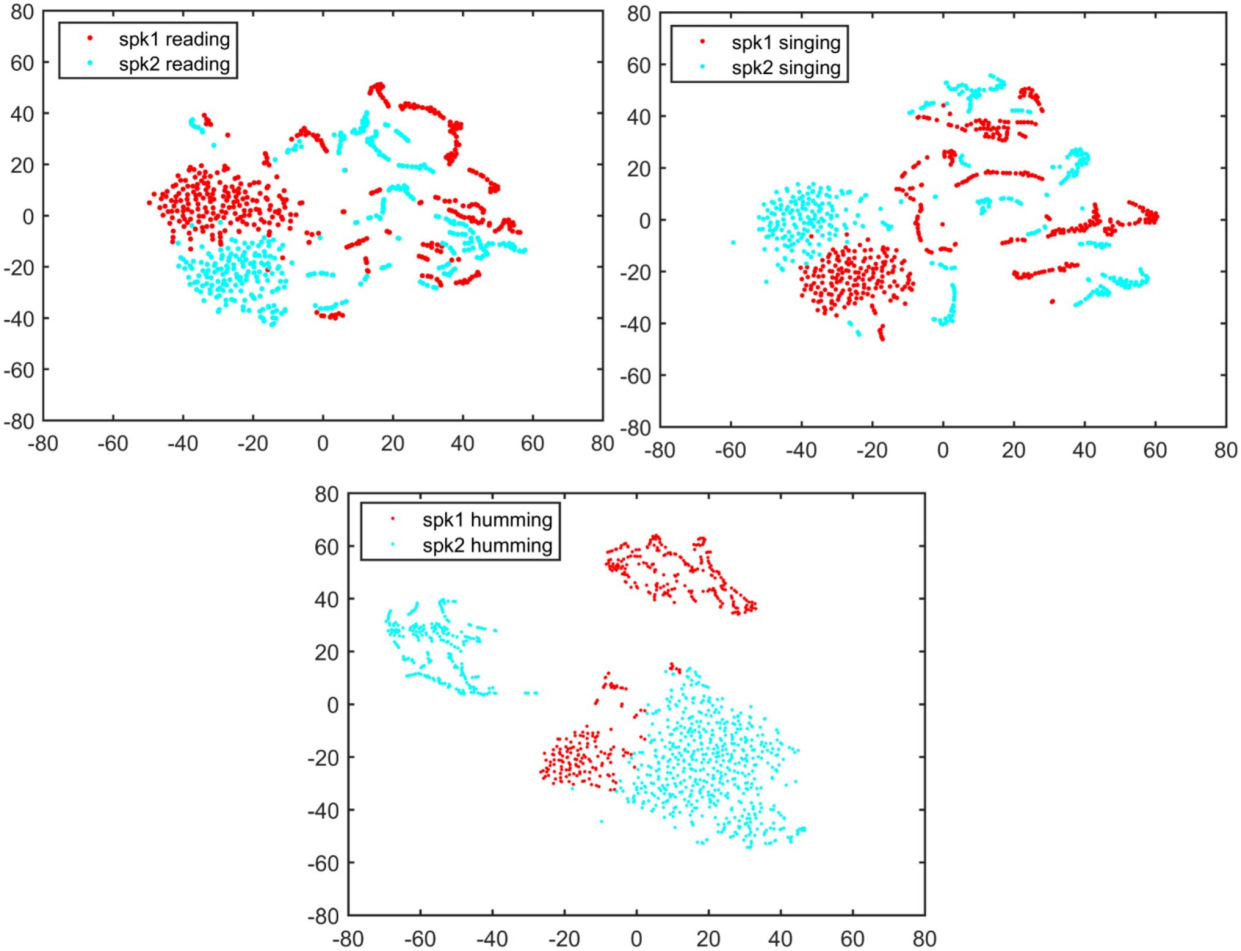
Two speaker’s feature space discrimination using t-SNE for each speaking style under text-dependent condition.

**Fig 3:**
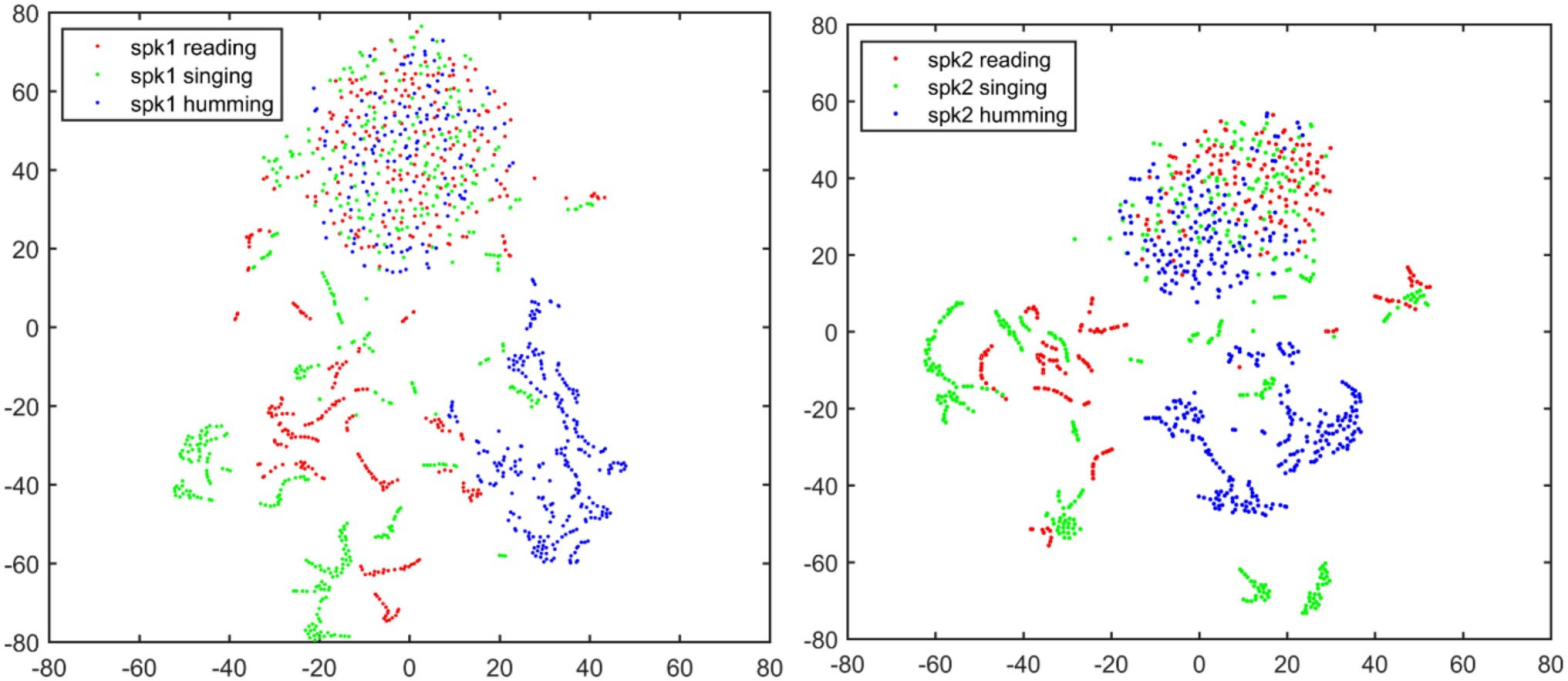
t-SNE visualization of two speakers’ features under text-independent condition. The texts are the same for each speaker with three speaking styles.

**Fig 4:**
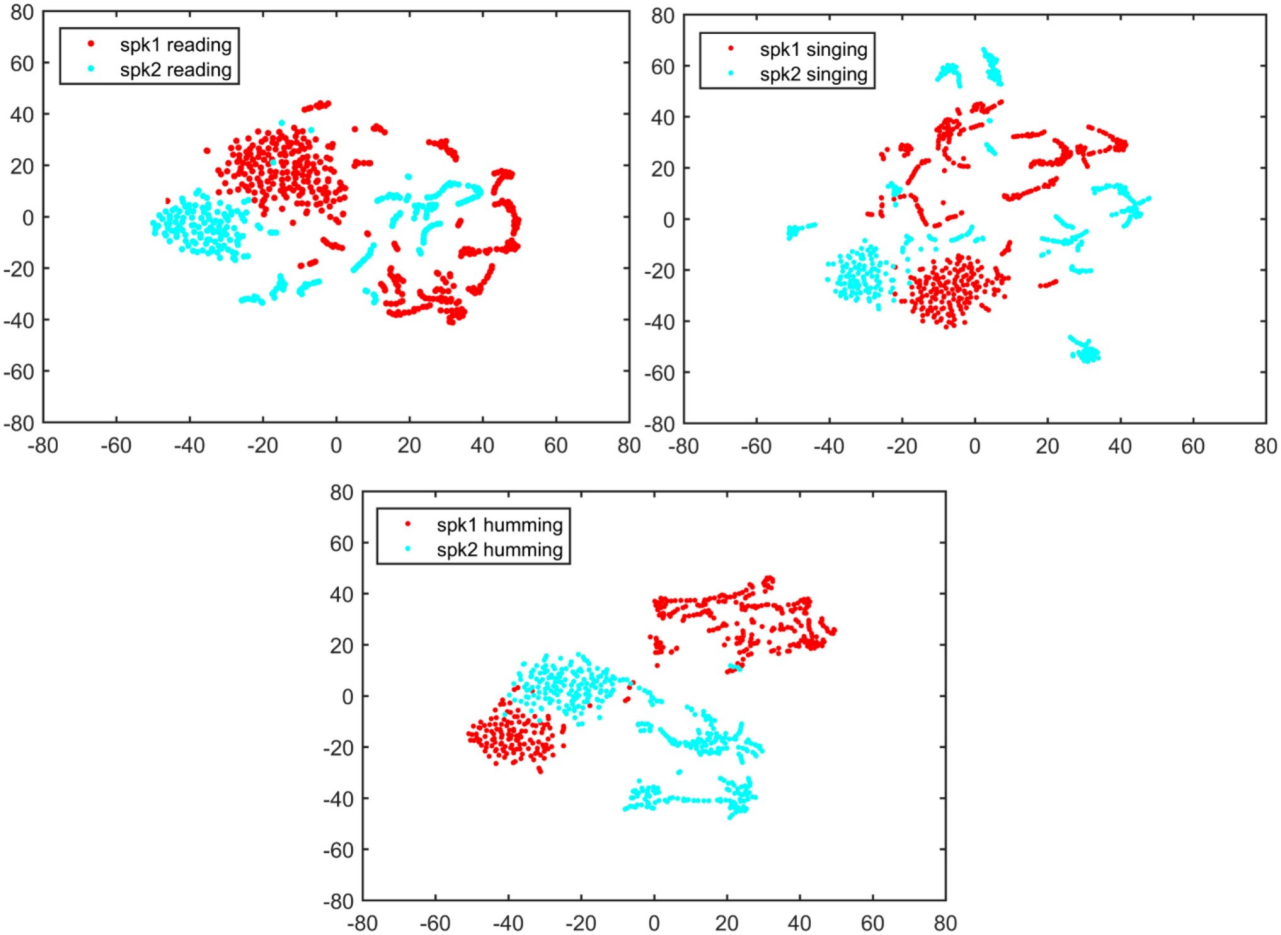
Two speaker’s feature space discrimination using t-SNE for each speaking style under text-independent condition. The texts are the same for each speaker with three speaking styles.

Figs 1 and 2 demonstrate the t-SNE visualization of two speakers’ features and between-speaker discriminations for text-dependent condition, while Figs 3 and 4 demonstrate the same visualizations for text-independent case. From both the Figs 1 and 3, we can see an overlap cluster in each subfigure, it indicates that the humming, singing and reading have part of common acoustic feature space of the same speaker, even most of their acoustic feature spaces reflect the same speaker identity clues in their own ways (non-overlap parts in Figs 1 and 3).

However, in the text-dependent case of Fig 2, we see that the humming achieves much better feature discrimination between two different speakers than the other two speaking-styles. And there is no significant gap between the reading and singing for speaker identity representation. For the text-independent case in Fig 4, we also see a much better feature discrimination between two speakers’s humming than their reading and singing. Therefore, we speculate that the humming speaking style may result in a much better ASV performance than the reading and singing. This observation is very consistent with the findings in the 70’s research that nasal sounds are more speaker discriminative [28]. The good discrimination of humming sounds may be due to the fact that the nasal tract is fixed whereas the vocal tract is more flexible [23, 24]. Also, different speakers may have different ability to control their vocal cord during the production of humming sounds [25].

## Speaker verification systems

### GMM-based system

According to statistical theory, enough Gaussian distributions can almost approximate any probability distribution. So as our previous work in [26], the conventional GMM are still used to model the speaker identity for each enrollment speaker. Assuming a *d*-dimensional input feature for each speech segment, the probability density function of GMM with *M* mixtures is as:

$$\begin{array}{c} p\left(x|\lambda \right)=\sum_{i=1}^{M}w_{i}g\left(x|\mu_{i},\Sigma_{i}\right) \end{array}$$ (1)

where λ = {*w*_*i*_, *μ*_*i*_, Σ_*i*_} is the GMM model, and *g*(*x*|*μ*_*i*_, Σ_*i*_) represents a *d*-variate Gaussian probability density function, with mean vector *μ*_*i*_, covariance matrix Σ_*i*_ and mixture weights *w*_*i*_ with $\Sigma_{i=1}^{M}w_{i}=1$. During the speaker enrollment, one GMM model will be built for each speaker using her/his enrollment speech recording. Given a set of acoustic features of each target speaker, the maximum likelihood (ML) criterion with a commonly used expectation maximization (EM) algorithm are used to estimate the parameters of the target speaker GMM model [1].

During the testing, given the *d*-dimensional feature vectors *X* = (*x*_1_, *x*_2_, …, *x*_*T*_) of test utterance with *T* frames, we compute the log-likelihood score on each target speaker model as:

$$\begin{array}{c} \Lambda \left(X\right)=\frac{1}{T}logp\left(X|\lambda \right)=\frac{1}{T}\sum_{t=1}^{T}log\sum_{i=1}^{M}w_{i}g\left(x_{t}|\mu_{i},\Sigma_{i}\right) \end{array}$$ (2)

These log-likelihood scores are then used to compare with a threshold to give the final speaker verification decision. More details of the GMM model training can be found in [1].

In the literature, comparing with directly apply EM algorithm to obtain the target speaker model, it’s well known that the MAP adaptation of GMM-UBM proposed by D.A Reynolds, et al [29] is a very effective approach to build target speaker GMM models, especially when there is not enough target speaker training data. In the MAP adaptation, an universal background model (UBM) trained with a large amount of background or irrelevant speaker data is required to be adapted to each target speaker through the MAP algorithm. However, in our work, we do not have enough abnormal vocalization corpus (including reading, singing and humming speaking styles) to train a good enough UBM model (abnormal vocalization UBM), because the total amount of RSH corpus is only around 2 hours (please refer to the section “RSH Corpus” for data collection details), and in order to construct a “reasonably large” ASV task to obtain more stable performance behavior, we used all of the RSH data to construct the ASV task, including both the target speaker training data and the test utterances for non-overlap test trials as shown in Table 2. Therefore, there is no RSH data that can be used to train an “abnormal vocalization UBM model”. And it worth noting that we cannot use any of the target speakers’ training data to train an UBM, because according to the MAP adaptation principle of GMM-UBM described in [29], an UBM model should be trained from large amount of background imposters’ or irrelevant speakers’ utterances that are not included in the ASV tasks. Therefore, in our experiments, we choose to train the small-size speaker-dependent GMM instead of using UBM-based MAP adaptation to obtain the target speaker model.

In fact, in our initial experiments, we also tried using easily available data (clean reading speech such as AISHELL-1 [30]) to train an UBM and then applied MAP to obtain the adapted target speaker model, however, as the observation from x-vector systems in paragraph 2 of section “Results with Single-speaking style”, all the adapted GMMs were heavily biased to the reading speaking style. The biased models make the speaker discrimination of singing and humming incapable of being reflected. Therefore, to avoid the heavy bias issue and emphasize the effects of individual speaking styles, in the below GMM-based experiments, we finally decide to directly apply EM instead of MAP to obtain the target speaker models.

### X-vector based system

With the rapid development of deep neural network technology in speaker recognition, compared with traditional generative models and the recently proposed end-to-end network models [31], DNN embedding is currently the most robust with almost the best generalization ability for speaker recognition. There are many types of DNN embeddings: DNN-ivector [8, 32], d-vector [10], J-vector [33], S-vector [34], etc. The principle of all of these vectors is to map a variable-length speech utterance to a discriminative fixed-dimensional space for speaker modeling using variety deep neural networks.

Besides the GMM-based framework, in this work, the state-of-the-art x-vector [9] ASV framework is also used to validate the effectiveness of multi-speaking style speech. The x-vector has been proved to be very effective and become the dominant technique for both TI and TD speaker verification [35]. We use the same x-vector embedding architecture as in [9]. To build an x-vector based ASV system, we need to train an x-vector extractor first, then given each speech segment, we can extract an x-vector from the well-trained extractor to represent the speaker identity. During the testing, the simple cosine distance between the x-vector of each test speech and the one of target speaker enrollment speech is computed as the final verification decision score. Normally, in order to improve the robustness and generalization ability of these x-vectors, a PLDA backend [3, 9] is applied to these x-vectors. Please refer to the work of [9] to obtain more details of the extractor training.

## Experiments and results

### Configurations

The 20-dimensional MFCCs and their delta and delta-delta dynamic features [36] are used to train both the GMM-based and x-vector systems. All of these features are extracted using a 25 ms hamming window with a 10 ms frame shift. An energy-based voice activity detection (VAD) is applied to remove the silence.

For the GMM-based ASV systems, 32 GMM mixtures with diagonal covariance are used in experiments. In fact, we have tried the mixtures from 8 to 1024 and the 32 mixtures GMM obtained the best and stable results. The x-vector architecture and system configurations are the same as used in our precious work of [26]. We still use the open-source speech corpus “AISHELL-2” [37] to train our x-vector extractor. It has 1000 hours of clean read-speech data with 1991 speakers. The PLDA-based backend [3, 9] is also trained using 178 hours of “AISHELL-1” corpus with 400 speakers [30]. The speaker recognition open source toolkit-ALIZE 3.0 [38] is used to build our GMM-based systems. For the x-vector based systems, the Kaldi [39] main branch recipe at https://github.com/kaldi-asr/kaldi/tree/master/egs/sre16/v2 is used for our system training. The performances are reported in terms of equal error rate (EER) [1], a verification error measure that gives the accuracy at decision threshold for which the probabilities of false rejection (miss) and false acceptance(false alarm) are equal.

In addition, because of the clean and high-quality RSH corpus, we have obtained extremely good results on both GMM-based and x-vector ASV systems. The EERs are less than 1% for all of the three speaking style speech. It is meaningless to compare these results under this idea condition. Therefore, unlike our previous work, in this study, we expect to see the performance behavior under real ASV tasks with background noises. So, in the experiments, we add noise into all of the target speaker enrollment and test speech utterances to simulate real noise environments. All the results presented in the following sections are the experiments with noise-added. For additive noise, we use the MUSAN dataset, which consists of over 900 noises and can be freely available from http://www.openslr.org. The MUSAN noises are added to the original speech at 0-15dB signal-to-noise ratio using the sox toolkit.

### Results with single-speaking style

In Table 3, we examine the difference between Mandarin singing, humming and normal reading speech for text-dependent ASV tasks using two different systems, the conventional GMM-based and the state-of-the-art x-vector based systems. From preliminary results in Table 3, it is clear that under the same text-dependent ASV task, the performance gap between singing, humming and normal reading speech is very large. In the conventional GMM-based ASV systems, we can see that the voiceprint information extracted from singing and humming speech has much stronger speaker discrimination than the one extracted from reading speech, especially for the female speakers. Such as, compared with the TD-GD female result of reading speech, the singing and humming have obtained a relative 24.6% and 41.3% EER reductions respectively. The large EER reduction obtained in humming is consistent with the observation from the t-SNE feature space in section “Speaker Discrimination in Feature Space”.

**Table 3:** EER% on the text-dependent ASV tasks, using three types of single-speaking styles recordings.

System	Task	Gender	Reading	Singing	Humming
GMM-based	TD-GD	Male	24.0	22.5	18.0
		Female	30.0	22.6	17.6
	TD-GI	All	25.0	19.1	16.9
x-vector based	TD-GD	Male	3.0	3.5	6.5
		Female	6.1	5.3	8.0
	TD-GI	All	3.9	4.1	5.2

However, in the recent x-vector systems, we do not achieved consistent performance gains by using the personalized singing and humming speech. For the TD-GD task, only singing speech obtained slightly better result (5.3%) than other two types of speech. The EERs from humming are much higher than the ones from reading and singing. As mentioned in the last paragraph of section “GMM-based System”, these non-consistent observations are similar to our findings from the results of initial GMM-UBM based systems. We guess that they may result from two aspects, one is the stronger acoustic information modeling ability of x-vector neural network extractor than the GMM. We speculate that given the same text, the individual speaker discriminative information in humming may not richer than in reading and singing speech when they meet a stronger information extractor. The other aspect is the AISHELL reading speech corpus we used to train the x-vector extractor and PLDA backend. The acoustic properties learned by the extractor and PLDA deviate far from the singing and humming speech. They are biased to the reading speech. The biased models make the speaker discrimination of singing and humming incapable of being reflected. Even we already have RSS and RSH, however, for a deep neural network or even an UBM, the total amount of training data is still not enough. We will re-validate the x-vector based systems in our future works, when enough multi-style speaking speech is available to train an unbiased deep x-vector extractor.

In addition, from Table 3, we see that the results of female are much worse than that of male for almost all speaking styles, either in the GMM-based, or the x-vector based systems. It indicates that females have bigger inter-speaker confusion than males. This may due to the fact that there is big physiologic and vocalization differences of female and males, female voices are more difficult to be distinguish in acoustics [40]. Moreover, from the literature, we find that many previous works have also obtained the consistent results with our work, their speaker verification results of female are also much worse than that of male [41, 42], so in many conventional ASV systems, the target speaker models are built in gender-dependent way [41, 43].

Since the most ASV tasks are gender-independent, for clear, we only report gender-independent results in next experiments. Table 4 presents the results on TI-GI ASV tasks for both GMM-based and x-vector based systems. It’s clear to see that all the EERs are much higher than the ones in Table 3, it tells us that the text-independent ASV task is more challenge than the text-dependent one. Different from observations on the text-dependent task, the performance gap between reading and singing speech are greatly reduced. Moreover, the flexible humming text introduced a much better speaker discrimination over the reading and singing. The ASV EERs are significantly reduced by using text-independent humming speech. Other observations are consistent as in Table 3.

**Table 4:** EER% on the TI-GI ASV tasks, using three types of single-speaking styles recordings.

System	Reading	Singing	Humming
GMM-based	34.9	31.7	21.5
x-vector based	16.8	17.4	9.5

### Results with cross-speaking style

Tables 5 and 6 demonstrate the performance behaviors on ASV cross-speaking style tasks for the GMM-based and x-vector system respectively. The diagonal numbers in both Tables are the same as in Tables 3 and 4. In the GMM-based systems, we see that the speaking-style mismatch between target speaker enrollment and test speech is very significant for the text-dependent ASV task. More than around absolute 10% EER increases are result from the cross-speaking mismatch. The performance gap between different cross-speaking tests on TI-GI system are much smaller. This may due to the challenging TI ASV task itself. In addition, both on the TD and TI tasks, we see a much smaller performance gap between reading and singing cross-testing. It indicates that these two speaking-styles are acoustic-similar at some extent. However, the cross-speaking tests with humming and reading, or humming and singing achieved near absolute 20% EER increases over the humming speaking-style matched condition.

**Table 5:** EER% on the GMM-based cross-speaking ASV tasks.

Task	Test	Reading	Singing	Humming
	Enroll			
TD-GI	Reading	**25.0**	30.4	38.0
	Singing	31.7	**19.1**	38.0
	Humming	38.4	37.6	**16.9**
TI-GI	Reading	**34.9**	37.2	40.0
	Singing	36.4	**31.7**	38.4
	Humming	39.2	38.8	**21.5**

**Table 6:** EER% on the x-vector based cross-speaking ASV tasks.

Task	Test	Reading	Singing	Humming
	Enroll			
TD-GI	Reading	**3.9**	14.1	32.8
	Singing	20.7	**4.1**	32.8
	Humming	34.3	32.1	**5.2**
TI-GI	Reading	**16.8**	20.2	33.0
	Singing	20.6	**17.4**	33.1
	Humming	34.4	33.3	**9.5**

In the x-vector based systems, the similar observations from cross-speaking tests have been obtained as in the GMM-based systems. However, it is interesting to find that, on the cross-speaking ASV tasks, the absolute performance of each x-vector based system is not significantly improved over the GMM-based system. Especially for these systems with humming cross-testing. Besides the x-vector extractor training data issue, this may also due to the fact that, both the conventional GMM and state-of-the-art x-vector ASV system are vulnerable to deal with the real cross-speaking ASV applications.

### Result with multi-speaking style data combination

In Table 7, we investigate the possibility of combining multi-speaking style data to improve the ASV systems. In our experiments, we keep the same test set as the experiments in above Tables, only three types of target speaker enrollment utterances are combined together. Specifically, in the GMM-based system, we directly concatenate three speaking style enrollment utterances together to build the target speaker GMM model. While in the x-vector based systems, for each target speaker enrollment, we first extract an x-vector for each reading, singing and humming utterance, and then average them to form a better speaker-level x-vector.

**Table 7:** EER% on both the GMM-based and x-vector based ASV tasks using multi-speaking style training data combination.

System	Task	Reading	Singing	Humming
GMM-based	TD-GI	26.3	21.7	22.0
	TI-GI	33.8	30.7	26.7
x-vector based	TD-GI	1.3	1.7	7.8
	TI-GI	11.2	11.8	11.3

The preliminary results in Table 7 show that, if the test speech is humming, the data combination in both GMM and x-vector based systems degraded the ASV performances. If the test speech is singing or reading, the multi-style data combination is very effective in the x-vector based system, such as, on the reading test set, the EER is reduced from 3.9% to 1.3%, and 16.8% to 11.2% for TD-GI and TI-GI task respectively; On the singing test set, the EER is reduced from 4.1% to 1.7%, and 17.4% to 11.8% for TD-GI and TI-GI task respectively. However, on the GMM-based system, the data combination only slightly improved or even worsen the performance for reading and singing test set. This may due to the weak speaker modeling ability of GMM than deep neural network based x-vector extractor.

### X-vector based cheating experiments

As shown in section “RSH Corpus”, the total amount of RSH corpus is only around 2 hours, so in order to construct a “reasonable large” ASV task to obtain more stable performance behavior, in Table 2, we used all of the RSH data to construct the ASV tasks, including both the target speaker training data and the test utterances for non-overlap test trials. Therefore, as discussed in the above sections, there is no other RSH data can be used as the in-domain development dataset to train an UBM or x-vector extractor. In addition, according to the principle of normal ASV system building, the development data for system training can not include any test utterances. That’s why we only obtain a reading-speech biased x-vector extractor in our above experiments.

However, it is interesting to see the behavior of an unbiased x-vector extractor for these multi-speaking style ASV tasks. Therefore, in this section, we present the results for a group of cheating experiments by using all of the RSH to train an unbiased x-vector extractor. Because the RSH data size is only around 2 hours, it is very difficult to train a very deep neural network, therefore, instead of using the 7 layers time-delay deep neural network (TDNN) architecture for the above biased extractor, here we reduce the TDNN to 4 layers, by removing the 3rd, 4th and the 7th layers, and we also reduce the dimension of hidden layers from 512 to 64.

Results in Table 8 are the EER performances for both single-speaking style TD-GI and TI-GI ASV tasks. Compared with the results from reading-speech biased x-vector extractor in Tables 3 and 4, these EERs are much larger because of the limited RSH corpus and the small-size TDNN. However, we note that the system performance behavior is very consistent with the one achieved from the GMM-based systems. The singing and humming speech contain much stronger speaker discrimination information than the reading speech. And the humming speech achieved the best results in both the text-dependent and text-independent ASV tasks.

**Table 8:** EER% on the single-speaking styles TD-GI and TI-GI ASV tasks, using the whole RSH to train the x-vector extractor.

System	Task	Reading	Singing	Humming
x-vector based	TD-GI	22.6	17.8	15.2
	TI-GI	26.5	23.7	16.7

The cheating results in Tables 9 and 10 should be compared with their corresponding results in Tables 6 and 7 for the x-vector based cross-speaking ASV tasks, and the single-speaking ASV task with multi-speaking style training data for the target speaker enrollment respectively. From Table 9, we achieved the same observation as in Table 6 for the cross-speaking ASV tasks, even these results are from an unbiased x-vector extractor. However, the observations in Tables 7 and 10 are very different, unlike the degraded performances for humming test set in Table 7, the performances in Table 10 are significantly improved for all the reading, singing and humming test sets over the ones in Table 8. It indicates that the multi-speaking style training data combination is very effective to improve the generalization ability for multi-speaking style ASV systems, the acoustic variability in different speaking style speech data has significant complementary speaker identity information.

**Table 9:** EER% on the x-vector based cross-speaking ASV tasks, using the whole RSH to train the x-vector extractor.

Task	Test	Reading	Singing	Humming
	Enroll			
TD-GI	Reading	**22.6**	25.6	32.0
	Singing	26.7	**17.8**	33.7
	Humming	32.2	33.0	**15.2**
TI-GI	Reading	**26.5**	29.2	33.0
	Singing	29.0	**23.7**	34.2
	Humming	33.3	33.7	**16.7**

**Table 10:** EER% on the x-vector based ASV tasks using the whole RSH to train the x-vector extractor, and multi-speaking style training data combination for the target speaker enrollment.

System	Task	Reading	Singing	Humming
x-vector based	TD-GI	19.8	16.7	17.3
	TI-GI	23.4	19.4	14.3

Although the results in this section are obtained from cheating experiments, it is still useful for us to see the ASV system behavior between a biased and unbiased x-vector extractor. In conclusion, both the GMM-based and unbiased x-vector based systems show that the speaker discriminative information in both humming and singing is much stronger than in the normal reading speech, and the humming achieves the best results. Furthermore, results also indicate that combining multi-speaking style speech for enrollment is very helpful to improve the ASV system performance.

## Conclusions

This study attempts to investigate how the multi-speaking styles affect the automatic speaker verification. A new ASV corpus-RSH has been designed and released freely in the Zenodo website for public research. This corpus has expanded our previous RSS corpus in two aspects, one is that RSH contains three speaking-styles, reading, singing and humming; The other is that, this new corpus can be used to design both the text-dependent and text-independent ASV experiments, either for single-speaking style or multi/cross-speaking style ASV tasks. Based on this corpus, we have examined the behavior of different speaking styles in both the feature space and the ASV system level. Experimental results show that, in both of the text-dependent and text-independent GMM-based and unbiased x-vector based systems, the humming shows the best speaker discriminative information than the singing, and the singing is better than the normal reading, although the observation is not consistent under a reading speech biased x-vector system.

Furthermore, the cross-speaking style in speaker enrollment and test stages is also studied. We find that both the GMM and x-vector are very vulnerable to deal with this issue. And in addition, we observe that combining the multi-speaking style training data together can significantly improve the ASV performances of x-vector based systems, although almost no performance gains have been obtained in the conventional GMM-based systems. Our future works will focus on developing better un-biased deep neural network based ASV system to exploit the multi-style training data and handing the cross-speaking issue between enrollment and test stages.
